# Molecular portraits revealing the heterogeneity of breast tumor subtypes defined using immunohistochemistry markers

**DOI:** 10.1038/srep14499

**Published:** 2015-09-25

**Authors:** Xiaofeng Dai, Yang Li, Zhonghu Bai, Xu-Qing Tang

**Affiliations:** 1 School of Biotechnology, Jiangnan University, Wuxi 214122, China; 2 School of Science, Jiangnan University, Wuxi 214122, China; 3 National Engineering Laboratory for Cereal Fermentation Technology, Jiangnan University, Wuxi 214122, China

## Abstract

Breast cancer is highly heterogeneous. The subtypes defined using immunohistochemistry markers and gene expression profilings (GEP) are related but not equivalent, with inter-connections under investigated. Our previous study revealed a set of differentially expressed genes (diff-genes), containing 1015 mRNAs and 69 miRNAs, which characterize the immunohistochemistry-defined breast tumor subtypes at the GEP level. However, they may convey redundant information due to the large amount of genes included. By reducing the dimension of the diff-genes, we identified 119 mRNAs and 20 miRNAs best explaining breast tumor heterogeneity with the most succinct number of genes found using hierarchical clustering and nearest-to-center principle. The final signature panel contains 119 mRNAs, whose superiority over diff-genes was replicated in two independent public datasets. The comparison of our signature with two pioneering signatures, the Sorlie’s signature and PAM50, suggests a novel marker, FOXA1, in breast cancer classification. Subtype-specific feature genes are reported to characterize each immunohistochemistry-defined subgroup. Pathway and network analysis reveal the critical roles of Notch signalings in [ER+|PR+]HER2− and cell cycle in [ER+|PR+]HER2+ tumors. Our study reveals the primary differences among the four immunohistochemistry-defined breast tumors at the mRNA and miRNA levels, and proposes a novel signature for breast tumor subtyping given GEP data.

Breast cancer is a heterogeneous disease accompanied by differences in clinical, molecular and biological features^1^, which creates a challenge for prognosis and treatment^2^. Traditionally, immunohistochemistry (IHC) markers together with clinicopathologic indexes are used to classify breast cancer and predict disease outcome^3^. Increasing number of IHC molecules have been identified to play critical roles in breast tumor subtyping, among which estrogen receptor (ER), progesterone receptor (PR) and human epidermal growth factor receptor 2 (HER2) are the most commonly used. Based on these molecular markers, breast cancer can be classified into four basic subgroups, i.e., [ER+|PR+]HER2− (positive ER and PR status, and negative HER2 status), [ER+|PR+]HER2+ (positive ER, PR and HER2 status), [ER−|PR−]HER2+ (negative ER and PR status, and positive HER2 status), [ER−|PR−]HER2− (negative ER, PR and HER2 status)^4^. Over a decade ago, gene expression profiling (GEP) has been applied to capture breast tumor heterogeneity and subtyping. Sørlie *et al.*^5^,^6^,^7^ firstly proposed the usage of “intrinsic” genes to classify breast tumors into four major subtypes, i.e., luminal A, luminal B, HER2 positive, basal-like, and the normal-like tumors in addition. Parker *et al.*^8^ developed a classifier composed of 50 genes, namely PAM50, to identify the four major intrinsic subtypes. Each of the four major IHC-defined subtypes corresponds to a basic GEP-defined subgroup. Luminal A and luminal B are roughly equivalent to [ER+|PR+]HER2− and [ER+|PR+]HER2+ tumors, respectively, though a small percentage of [ER+|PR+]HER2− tumors with Ki67 positivity are reported to belong to the luminal B subtype^9^. HER2 positive tumors refer to [ER−|PR−]HER2+ despite the different methods used on HER2 assessment. The [ER−|PR−]HER2− (also named triple negative tumors, TNP) subtype is mainly composed of basal-like tumors, which is highly heterogeneous including at least claudin-low^10^, metaplastic breast cancer^11^ and interferon-rich tumors^12^ in addition to core basal tumors as demonstrated by the accumulated evidence. Dai *et al.*^4^ have reported a set of diff-genes, which is composed of 1015 mRNAs and 69 miRNAs that are differentially expressed among the four IHC-defined breast tumor subtypes. Though the diff-genes well capture the differences among these subtypes and could be used for GEP-based subtyping in principle, the large amount of genes included in the gene set may not be feasible for clinical use. Also, only a small subset of genes are, in general, relevant and the useful information may be masked by the other genes that are either redundant or noisy. We are thus inspired to identify the representatives of the diff-genes, aiming at obtaining the best subtyping accuracy with the most succinct number of genes. Subtype-specific feature genes are also revealed to characterize the differences among these IHC-defined subtypes. Network and pathway analysis were conducted to uncover the interconnections and functional roles of these signature genes. Our study reveals the core differences explaining the heterogeneity of the four basic subtypes defined using ER, PR and HER2 status at the mRNA and miRNA expression levels. It bridges the gap between IHC and GEP in differentiating breast tumor subtypes and could be used for subtyping of such tumors given gene expression data.

## Results

### Identification and performance assessment of the signature genes

**Figure 1:**
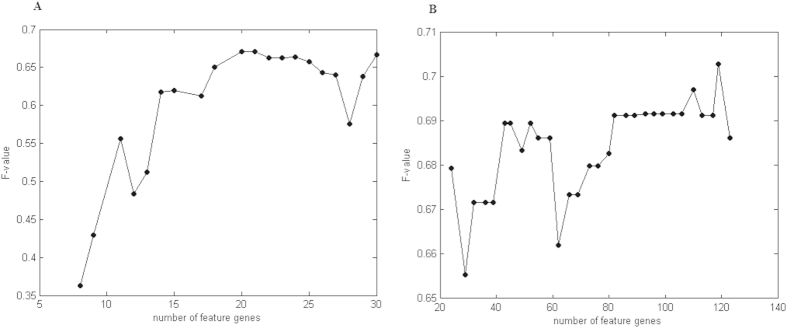
Selection of the number of (**A**) miRNA and (**B**) mRNA feature genes.

The diff-genes presented in^4^ are the differentially expressed genes among four breast tumor subtypes defined using ER, PR and HER2 status. It is comprised of 1015 mRNAs and 69 miRNAs, which were reduced to 119 mRNA and 20 miRNA, namely the feature genes (Supplementary Table 1 and Supplementary Table 2), by maximizing the F-values in this paper (Fig. 1). The number of feature genes was determined for each breast tumor subtype (Supplementary Figure 1). Altogether, 13 (out of 379), 19 (out of 65), 16 (out of 152), 18 (out of 777) feature mRNAs, 10 (out of 30), 3, 5, 11 (out of 58) feature miRNAs were selected for [ER+|PR+]HER2−, [ER+|PR+]HER2+, [ER−|PR−]HER2+, [ER−|PR−]HER2− tumors, respectively (Supplementary Table 3).

**Figure 2:**
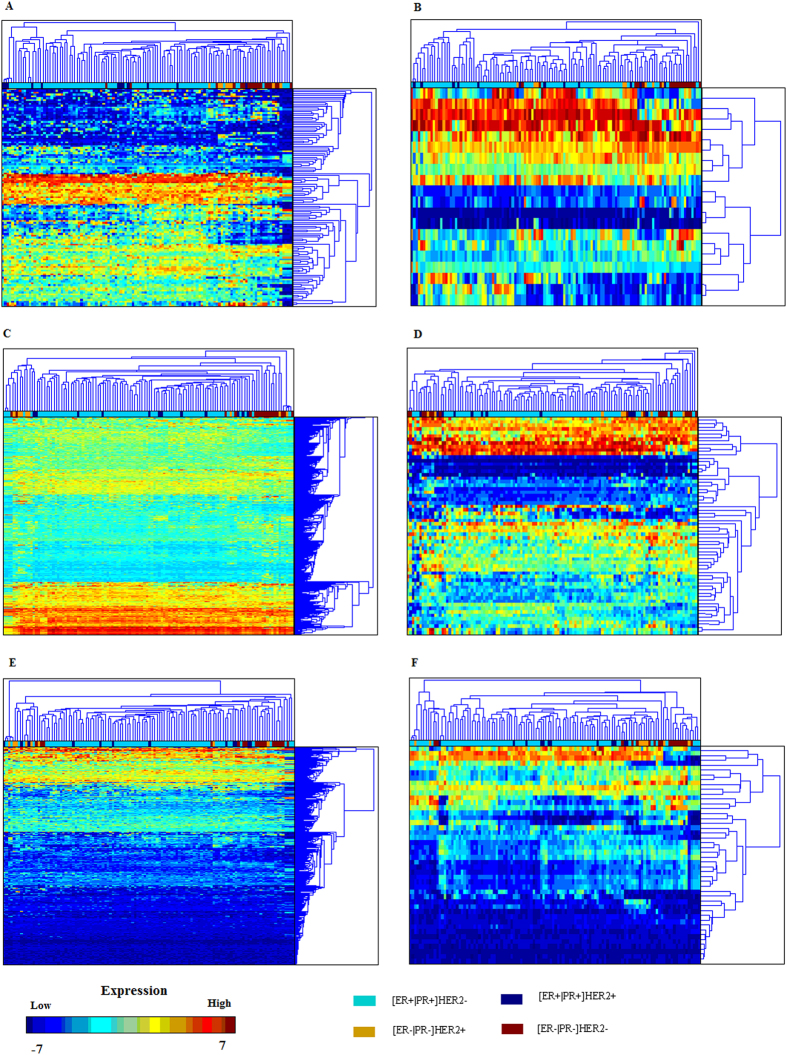
Hierarchical clustering of breast tumor samples in HEBCS using (**A**) mRNA feature genes (the signature), (**B**) miRNA feature genes, (**C**) mRNA diff-genes, (**D**) miRNA diff-genes, (**E**) Sorlie’s signature, (**F**) PAM50 genes. Molecules shown in red (increased expression) and green (decreased expression) identified in different sets of genes.

The performance of these feature genes in subtyping tumor samples was compared with the original diff-genes^4^ using the HEBCS dataset (Fig. 2). The clustering accuracies (measured by F-value and Rand index) were summarized in Table 1, with the patterns displayed in Fig. 2. The F-value and Rand-index using the mRNA feature genes are higher than the corresponding diff-genes in the HEBCS dataset (Table 1: F-value 0.7029 vs. 0.6599; Rand-index 0.7272 vs. 0.6577, Fig. 2A vs. Fig. 2C), and so as to miRNAs (Table 1: F-value 0.6712 vs. 0.5682; Rand-index 0.6898 vs. 0.5, Fig. 2B vs. Fig. 2D). The mRNA feature genes have higher accuracy than that of the miRNA feature genes (Table 1: F-value 0.7029 vs. 0.6712; Rand-index 0.7272 vs. 0.6898, Fig. 2A vs. Fig. 2B). The performance of the unified mRNA and miRNA feature genes (‘the signature’) has the same F-value and Rand-index as the mRNA feature genes. We, thus, include only mRNAs in the signature to make it as concise as possible. The performance improvement of the signature over mRNA diff-genes was replicated using GSE22220 (Table 1: F-value 0.8449 vs. 0.7084; Rand-index 0.7454 vs. 0.6175, Supplementary Figure 2A vs. Supplementary Figure 2B). The performance of the signature was compared with the Sorlie’s signature^5^, the first widely accepted gene list differentiating breast tumor subtypes (Table 1: F-value 0.7029 vs. 0.63; Rand-index 0.7272 vs. 0.5981, Fig. 2A vs. Fig. 2E), and PAM50, the most well-known gene panel for GEP subtyping (Table 1: F-value 0.7029 vs. 0.618; Rand-index 0.7272 vs. 0.6003, Fig. 2A vs. Fig. 2F), using HEBCS. Such performance superiority over the Sorlie’s signature (Table 1: F-value 0.8449 vs. 0.683; Rand-index 0.7454 vs. 0.5305, Supplementary Figure 2A vs. Supplementary Figure 2C) and PAM50 (Table 1: F-value 0.8449 vs. 0.7316; Rand-index 0.7454 vs. 0.6364, Supplementary Figure 2A vs. Supplementary Figure 2D) was also observed for the signature using GSE22220. Similar clustering accuracies were obtained for the signature genes, the Sorlie’s signature and PAM50 using the TCGA dataset (Table 1, Supplementary Figure 3).

The feature mRNAs and miRNAs (feature genes identified by using the diff-genes of all subtypes) were compared with the unified subtype-specific feature mRNAs and miRNAs (unified genes containing the feature genes identified from the diff-genes of each subtype). Out of the 119 mRNA feature genes and 62 unified subtype-specific feature mRNAs, 8 overlapped; and out of the 20 miRNA feature genes and 25 unified subtype-specific feature miRNAs, 9 overlapped. These overlapping genes, as listed in Table 2, might be the key molecules differentiating breast tumor subtypes.

### Pathway and disease analysis of the signature genes

Several cancer core pathways were found enriched in the signature genes, miRNA targets, subtype-specific feature genes or their union (Supplementary Table 4). In particular, cell adhesion molecules including VCAN, ALCAM, CLDN11, CLDN8, and CD6, were enriched in the signature genes (p = 0.004). The unified subtype-specific genes were present in the p53 pathway (p = 0.024). The targets of the miRNA feature genes were mostly involved in cell cycle (p = 0.03), mTOR (p = 0.043) and VEGF (p = 0.044) signalings. Among the four IHC-defined subtypes, genes of [ER+|PR+]HER2+ were enriched in DNA replication (p = 0.026), Notch signaling (p = 0.034) and the TGFβpathway (p = 0.056). We also checked the diseases relevant to the signature genes and feature genes of each subtype, with various cancers significantly enriched especially for the [ER−|PR−]HER2+ subtype (Supplementary Table 5).

**Figure 3:**
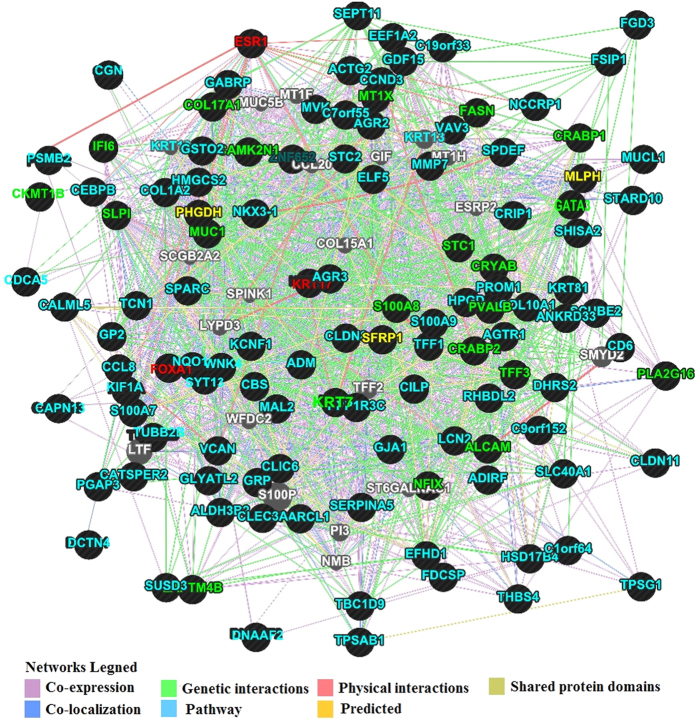
Network of the signature genes constructed using GeneMANIA. Red colored genes are shared between the signature, the Sorlie’s signature and PAM50 genes; yellow colored genes are shared between the signature and the Sorlie’s signature; green colored genes are shared between the signature and PAM50 genes.

Genemania was used to study the networks of the signature genes (Fig. 3), and the subtype-specific feature genes (Supplementary Figure 4). The sum of different links of the subtype-specific feature genes and the signature genes were summarized in Table 3. The feature genes of [ER+|PR+]HER2− tumors are involved in many known pathways and harbor many physical interactions. Those of [ER+|PR+]HER2+ tumors have the most shared protein domains, The feature genes of [ER−|PR−]HER2+ tumors are enriched by co-expressed genes, and [ER−|PR−]HER2− specific genes have the most co-localized genes among others. Genetic interaction is equally common among the feature genes of [ER+|PR+]HER2−, [ER+|PR+]HER2+ and [ER−|PR−]HER2− tumors, except for the [ER−|PR−]HER2+ subtype where the genetic interaction is rare. Co-expression is the most common among other interactions in the signature genes.

These signature genes are densely connected, among which several, such as ESR1, FOXA1, NQO1, GATA1, ALDH3B2, keratins, are well-known players driving the heterogeneity and carcinogenesis of breast tumors.

## Discussion

The mRNA and miRNA feature genes perform better than the original diff-genes reported in^4^ in differentiating the four IHC-defined tumor subtypes using HEBCS (Table 1: F value 0.7029 vs. 0.6599 for mRNA, 0.6712 vs. 0.5682 for miRNA; Rand Index p = 0.7272 vs. 0.6577 for mRNA, p = 0.6898 vs. 0.5 for miRNA), indicating that irrelevant genes have been efficiently removed from the signature which add little information but noise. MiRNAs perform less accurately than mRNAs, and do not contribute additional information to the signature on top of mRNAs. This, on one hand, may be caused by the complex and indirect influences of miRNAs on the phenotypic differences among breast tumor subtypes and, on the other hand, suggests the same pathways involved by the feature miRNAs and mRNAs (i.e., the targets of miRNAs share the same signaling with mRNAs). Actually, none of the validated miRNA targets overlaps with the signature mRNA genes, and so as to their enriched pathways which were retrieved from “KEGG Mapper—Search&Color Pathway” with the default parameter setting (Supplementary Table 4). KEGG database collects manually drawn pathway maps representing our current knowledge on molecular interactions and reaction networks. These seemingly inconsistent results imply that these genes, though being different and annotated to different KEGG pathways, may be involved in the same or alternative signaling with novel functional roles to be discovered.

The presented signature outperforms the Sorlie’s signature^6^ (which pioneers the field using gene expression profiling for breast tumor subtyping, Fig. 2E) and PAM50 genes (which is commonly applied for GEP-based breast tumor subtyping, Fig. 2F), with increased accuracy and moderate number of genes included as tested using HEBCS data (Table 1). The superiority of the signature over the diff-genes, Sorlie’s signature and PAM50 was replicated using GSE22220, demonstrating the generality and correctness of our observations. However, no significant difference regarding the classification accuracy was observed among the signature, diff-genes, the Sorlie’s signature and PAM50 using TCGA. This indicates that GEP-based clustering accuracy, though dominated by the genes included in the signature, is affected by the gene expression levels assessed, and the performance of the signature is at least as good as the Sorlie’s signature and PAM50.

Among the genes included in the signature, 25 and 6 are in common with the Sorlie’s signature and PAM50 genes, respectively, among which 3 are shared among all three datasets (Supplementary Table 6, Supplementary Figure 5). Several overlapping genes especially the ones present in all datasets (ESR1, FOXA1, KRT17) are known to play critical roles in the subtyping and carcinogenesis of breast tumors. For example, ESR1 is a discriminative factor between ER positive and ER negative tumors that mediates the biological effects of estrogens through direct binding to the estrogen response elements (EREs) of the target genes^13^; FOXA1 is associated with the methylation of the promoter of tumor suppressor genes and thus suggested as a potential demethylation target for the prevention and treatment of breast cancer^14^; cytokeratins such as KRT17 and KRT7 are basal markers and known to be up-regulated in circulating tumor cells^15^; and GATA3 is a transcriptional activator highly expressed in the luminal epithelial cells of the breast and lowly expressed in invasive carcinomas^16^, whose low expression is associated with ER negativity, PR positivity and HER2 over-expression^17^. Among the three genes shared by all signatures, two have already been applied for tumor subtyping, i.e., ESR1 is the primary marker classifying breast tumors into ER positive and ER negative subgroups and KRT17 plays crucial roles in differentiating the basal-like subtype from the other triple negative tumors, indicating that FOXA1 may be a novel immunohistochemistry marker for breast tumor classification.

The feature genes, selected from the unified diff-genes, have 8 mRNAs (6.7% of mRNA feature genes) and 9 miRNAs (45% of miRNA feature genes) overlapping with the unified subtype-specific feature genes (Table 2). The relatively small percentage of genes selected using both methods suggests the high heterogeneity of breast tumors and that these overlapping ones may play the key roles in distinguishing breast tumor subtypes. Most of these overlapping mRNA genes and miRNA targets are known to play critical roles in cancers or tumor cell lines. For example, ALCAM^18^,^19^,^20^ is associated with breast cancer migration and progression; GRP^21^,^22^ has mitogenic effects on some human breast cancer cell lines; SPARCL1^23^,^24^ is relevant to aggressive and invasive tumors and drives disease recurrence of prostate cancers; DHRS2^25^ encodes for Hep27 that is part of the molecular pathway regulating cell cycle and apoptosis in osteosarcoma and MCF7 breast cancer cells; CAMK2N1 plays a tumor suppressive role in prostate cancer and is suggested as a biomarker and therapeutic target of such tumors^26^. Has-miR-33b is known to target genes involved in cancer pathways such as MAPK, Wnt and Nf-kB signalings^27^. A direct target of has-miR-184, SND1, is suggested as a therapeutic target for malignant glioma^28^. Has-miR-135a/b modulate apoptosis via targeting MCL1 in lung cancer cell lines^29^. Interestingly, hsa-miR-135a and hsa-miR-135b share the same set of mRNA targets and play crucial roles in distinguishing breast tumors by ER positivity^4^, suggesting their non-redundant roles in distinguishing ER positive and ER negative breast tumors. Furthermore, hsa-miR-135b is characteristic of [ER−|PR−]HER2− tumors while has-miR-135a symbolizes the [ER+|PR+]HER2+ subtype in addition to [ER−|PR−]HER2−, implying an underlying connection between [ER+|PR+]HER2+ and [ER−|PR−]HER2− tumors, which are both aggressive.

The networks of the subtype-specific feature genes reveal the hub components representing each of these IHC-defined subtypes. NOTCH1, a key component present in [ER+|PR+]HER2− tumors, symbolizes the importance of Notch signaling in such cancers, which is an evolutionarily conserved mechanism that mediates communications between cells^30^. CDKN2A could induce cell cycle arrest in G1 and G2 phases^31^, whose presence in [ER+|PR+]HER2+ tumors suggests the representative roles of cell cycle signaling on tumors of this subtype.

## Conclusion

By reducing the dimensionality of the differentially expressed genes among IHC-defined subtypes presented in^4^, we report a 119-gene signature that captures the characteristics of these subtypes with improved accuracy and reduced number of genes. The feature genes of each subtype, including both mRNAs and miRNAs, are also presented, which explain the heterogeneity of the four basic IHC-defined subtypes. Comparison of our signature with the Sorlie’s signature and PAM50 suggests the crucial roles played by FOXA1 in breast cancer classification. Network analysis reveals the critical roles of Notch signaling in [ER+|PR+]HER2− and cell cycle in [ER+|PR+]HER2+. We present a set of signature genes rather than a tumor subtyping tool here, which better captures the differences among breast cancer subtypes than the genes included in the Sorlie’s signature and PAM50. It could be made available for breast tumor subtyping by relating a given sample to the centroid of each subtype determined using the expression of the signature genes from the training data, which would be our next step. As a reduced gene set of the diff-genes from^4^, the signature inherits the advantages of diff-genes. It bridges the gap between immunohistochemistry markers and gene expression profiling in breast tumor subtyping in addition to its integration of information at mRNA and miRNA levels. On top of that, the signature improves the subtyping accuracy and reduces the experimental cost, which better explains the heterogeneity of breast cancer and avails in the diagnosis of breast cancer patients as compared with the diff-genes reported in^4^.

## Material and Method

### Materials

The three public data sets employed in^4^ for diff-gene discovery, i.e., HEBCS, GSE22220, and TCGA were used in this study to identify and validate the signature genes.

HEBCS is composed of the mRNA (GSE24450) and miRNA (GSE43040) data from the GEO database^32^. This dataset harbors 24660 mRNAs (Illumina HumanHT-12_V3 Expression BeadChips) and 1104 miRNAs (IlluminaHumanMI_V2 BeadChips) for 183 primary breast tumor samples from the department of Oncology of the Helsinki University Central Hospital (HUCH) and department of Surgery^4^,^33^. The samples were grouped into four subtypes, i.e., [ER+|PR+]HER2−, [ER+|PR+]HER2+, [ER−|PR−]HER2+ and [ER−|PR−]HER2−, based on the status of ER, PR and HER2^4^. 1015 mRNAs and 69 miRNAs were identified differentially expressed among the four IHC defined subgroups.

GSE22220 consists of mRNA (GSE22219) and miRNA (GSE22216) data from GEO^32^. GSE22219 contains 24332 probes (Illumina Human Ref-8_V1 expression Bead Chips) for 216 patients, and GSE22216 contains 734 probes (Illumina HumanMI_V1 BeadChips) for 207 samples. These samples were grouped into ER+ and ER− tumors in^4^ based on its available IHC information.

TCGA data (level 3) was retrieved from the TCGA portal at http://tcga.cancer.gov/dataportal, which contains 17814 mRNAs (Agilent 244 K Custom Gene Expression G4502A-07-3) for 451 samples and 1046 miRNAs (IlluminaGA_miRNASeq) for 315 patients^4^. These primary solid tumor samples were classified into the four IHC-characterized subtypes as defined in the HEBCS data.

All datasets were pre-processed following instructions in^4^.

## Methods

### Hierarchical Clustering and accuracy assessment

Hierarchical clustering (HC) was applied to identify samples sharing similar expression levels according to a given set of genes. In the iterative process of HC, each sample is a point in a |G1| dimensional space, and all samples are clustered based on a certain similarity measure and the distance of these genes as measured according to their expression levels. The average linkage clustering algorithm was employed due to its efficiency in analyzing differential expression among samples.

Two well-known external evaluation indexes, i.e., Rand index and F-value^34^, were applied to assess the clustering accuracy provided with the knowledge on the ground-truth of the data structure.

Rand index considers the relationship between pairwise samples. Define the original and clustered set are *U* and *V*, respectively, there are four situations considering sample pair analysis, i.e.,
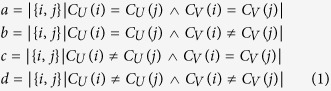
Based on the consistency and deviation, the Rand index is defined as
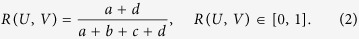
F-measure applies the concept of ‘precision’ and ‘recall’ from information retrieval here. They are defined as 
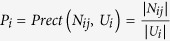
, 
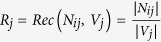
, where *N*_*ij*_ is the intersection set between classes *U*_*i*_ and *V*_*j*_, and 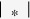 (* represents *U*_*i*_, *V*_*j*_ and *N*_*ij*_, respectively) denotes the number of the elements in each of these sets. F-value is determined by
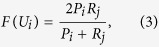
where 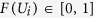.

### Signature gene identification

It is assumed that samples sharing similar expression profiles of the genes characterizing their heterogeneity (namely the signature genes) are likely to form a subtype that is phenotypically distinct from the other samples. To remove the irrelevant genes masking the roles of the signature genes in differentiating breast tumor subtypes and make the gene panel as succinct as possible, two steps were applied to the diff-genes^4^ which were differentially expressed among IHC-defined subgroups.

First, determine the number of feature genes (*N*(*C*_*r*_)) for each cluster by the cluster cohesiveness (which measures the closeness of a cluster). The cohesiveness of a class is given by
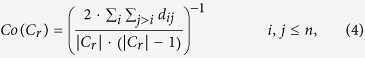
where 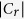 denotes the number of samples in class *C*_*r*_, and *d*_*ij*_ denotes the distance between 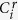 and 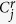 where the Euclidean distance is applied. The cohesiveness index reflects the similarity of the gene expression profiles within a class with a positive correlation. The number *N*(*C*_*r*_) of feature genes in class *C*_*r*_ is determined by maximizing the F-value according to
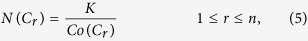
where *K*(*K* > 0) is the cohesive strength, and min(*K*) = 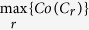 as each group has at least one signature gene. *N*(*C*_*r*_) and *Co*(*C*_*r*_) are negatively correlated as the more diverse a gene cluster is the more genes are needed to characterize^34^.

Second, select the signature genes for each cluster based on the nearest-to-center principle. Genes in class *C*_*r*_ could be divided into *N*(*C*_*r*_) subclasses using HC. The center *Cen*(*C*_*ri*_) of the subclass *C*_*ri*_ can be given by
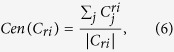
where 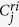 means that gene *j* belongs to the subclass *C*_*ri*_ of class *C*_*r*_. One feature gene is selected from each subclass by
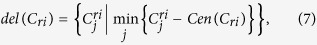
where *del*(*C*_*ri*_) is the agent selected from subclass *C*_*ri*_ that is closest to the fictitious center *Cen*(*C*_*ri*_). The representative genes for each subtype or a particular type of data are called the feature genes, and the final gene panel selected for characterizing breast tumor heterogeneity and subtyping is named ‘the signature’.

**Figure 4:**
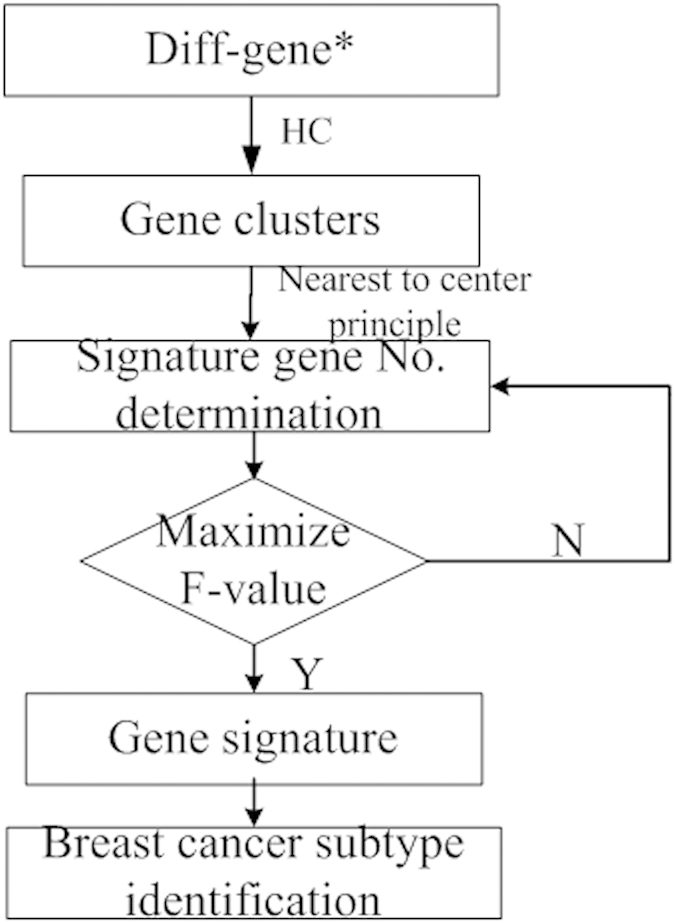
The process for signature gene identification. Genes marked by ^*^are taken from^4^.

The process for signature gene identification is illustrated in Fig. 4.

### Network and pathway analysis using gene signature

**Figure 5:**
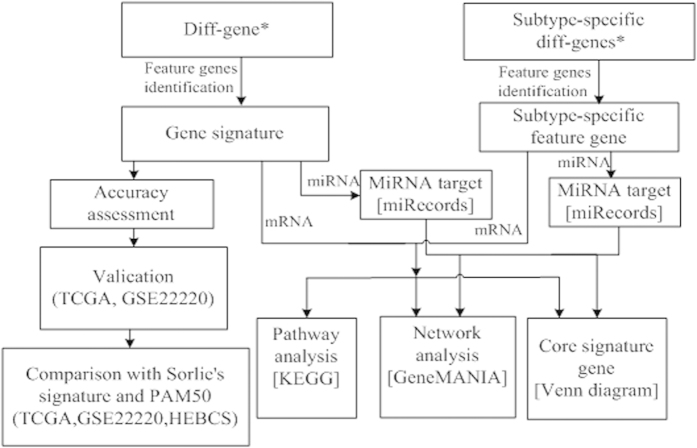
Work flow of the whole process deciphering the heterogeneity of breast tumor subtypes. Genes marked by ^*^are taken from^4^. Tools used in the analysis are shown in the square brackets, and the datasets used are listed in the round brackets.

To investigate the intrinsic heterogeneity of breast cancer, metabolic pathway and network analysis were applied to the obtained signature genes. MiRecords^35^, a resource integrating experimentally validated miRNA targets having systematic documentation of experimental support and predicted miRNA targets produced by 11 established prediction algorithms (predicted algorithms = 4), was used to find the targets of the feature miRNAs. DAVID^36^ (similarity term overlap = 4; similarity threshold = 0.85; group members = 3; multiple linkage threshold = 0.5 and EASE = 1) and KOBAS^37^(statistical method is hypergeometric test/Fisher’s exact test; FDR correction method is Benjamini and Hochberg; small term cutoff default = 5 ) were used to interpret the enrichment of gene ontology, metabolic pathway and relevant disease of these feature mRNAs and miRNA targets. The gene network was constructed using GeneMANIA^38^ (co-expression, co-localization, genetic interactions, pathway, physical interactions, predicted and shared protein domains were selected; automatically selected weighting method was used) to further elucidate the functional roles of the feature genes and the characteristics of each subtype. The whole process for identifying the signature genes and deciphering the heterogeneity of breast cancer subtypes is illustrated in Fig. 5.

## Additional Information

**How to cite this article**: Dai, X. *et al.* Molecular portraits revealing the heterogeneity of breast tumor subtypes defined using immunohistochemistry markers. *Sci. Rep.* **5**, 14499; doi: 10.1038/srep14499 (2015).

## Supplementary Material

Supplementary Information

## Figures and Tables

**Table 1:** Comparison of clustering accuracy between feature genes, diff-genes and well-known signature genes using different datasets.

Dataset	Gene	Dimension	F-value	Rand-index	Purpose
	miRNA diff-genes	69	0.5682	0.5	Identification
	miRNA feature genes	20	0.6712	0.6898	Identification
	mRNA diff-genes	1015	0.6599	0.6577	Identification
HEBCS	mRNA feature genes (the signature)	119	0.7029	0.7272	Identification
	The unified subtype-specific genes	139	0.7029	0.7272	Identification
	Sorlie’s signature	456	0.63	0.5981	Comparison
	PAM50	50	0.618	0.6003	Comparison
	mRNA diff-genes	1015	0.7084	0.6175	Validation
GSE22220	mRNA feature genes (the signature)	119	0.8449	0.7454	Validation
	Sorlie’s signature	456	0.683	0.5305	Comparison
	PAM50	50	0.7316	0.6364	Comparison
	mRNA diff-genes	1015	0.7225	0.7044	Validation
TCGA	mRNA feature genes (the signature)	119	0.7237	0.7032	Validation
	Sorlie’s signature	456	0.7189	0.7028	Comparison
	PAM50	50	0.7304	0.7068	Comparison

**Table 2:** Overlapping genes between the feature genes and the unified subtype-specific genes.

mRNA			miRNA		
*ALCAM*	*CAMK2N1*	*EFHD1*	*HS_239*	hsa-miR-130b*	*hsa-miR-135b*
*SPARCL1*	*DCTN4*	*GRP*	hsa-miR-101*	*hsa-miR-33b*	*hsa-miR-135a*
*C19orf33*	*DHRS2*		*hsa-miR-184*	*hsa-miR-521*	*hsa-miR-411*

**Table 3:** Link properties in the network of signature genes and subtype-specific feature genes.

Links	[ER+|PR+] HER2−	[ER+|PR+] HER2+	[ER−|PR−] HER2+	[ER−|PR−] HER2−	The signature
Co-expression	54	40	86	9	1141
Co-localization		8		44	133
Genetic interaction	20	31		26	310
Pathway	48				8
Physical interaction	47	6			25
Shared-protein domain		95	7		66
Total links	185	180	93	79	1694
