# Supplementary material for: Molecular portraits revealing the heterogeneity of breast tumor subtypes defined using immunohistochemistry markers
Source: Sci Rep. 2015 Sep 25;5:14499. doi: 10.1038/srep14499 (PMC4585919; doi:10.1038/srep14499)
Supplement: Supplementary Information [file srep14499-s1.pdf]

Molecular portraits revealing the heterogeneity of breast tumor subtypes defined using immunohistochemistry markers

Xiaofeng Dai[1, 3], Yang Li[2], Zhonghu Bai[1, 3], Xuqing Tang[2]

1: School of Biotechnology, JiangNan University, Wuxi 214122, China
2: School of Science, JiangNan University, Wuxi 214122, China
3: National Engineering Laboratory for Cereal Fermention Technology, Jiangnan University, Wuxi 214122, China

Supplementary Information

Supplementary figure legends

Supplementary Figure 1- The selection of the number of feature mRNAs.

Supplementary Figure 2- Hierarchical clustering of breast tumor samples in GSE22220.

Supplementary Figure 3- Hierarchical clustering of breast tumor samples in TCGA.

Supplementary Figure 4- Network of subtype-specific feature genes constructed by GeneMANIA.

Supplementary Figure 5- Venn diagram illustrating the relationship among the signature, the Sorlie's signature and PAM50 genes.

Supplementary figures

Supplementary Figure 1- The selection of the number of feature mRNAs. (A) [ER+|PR+]HER2-, (B) [ER+|PR+]HER2+, (C) [ER-|PR-]HER2+, (D) [ER-|PR-]HER2- and miRNAs for (E) [ER+|PR+]HER2- and (F) [ER-|PR-]HER2-. [ER+|PR+]HER2+ and [ER-|PR-]HER2+ were exempt from feature miRNA selection due to the few diff-genes included in these subtypes.

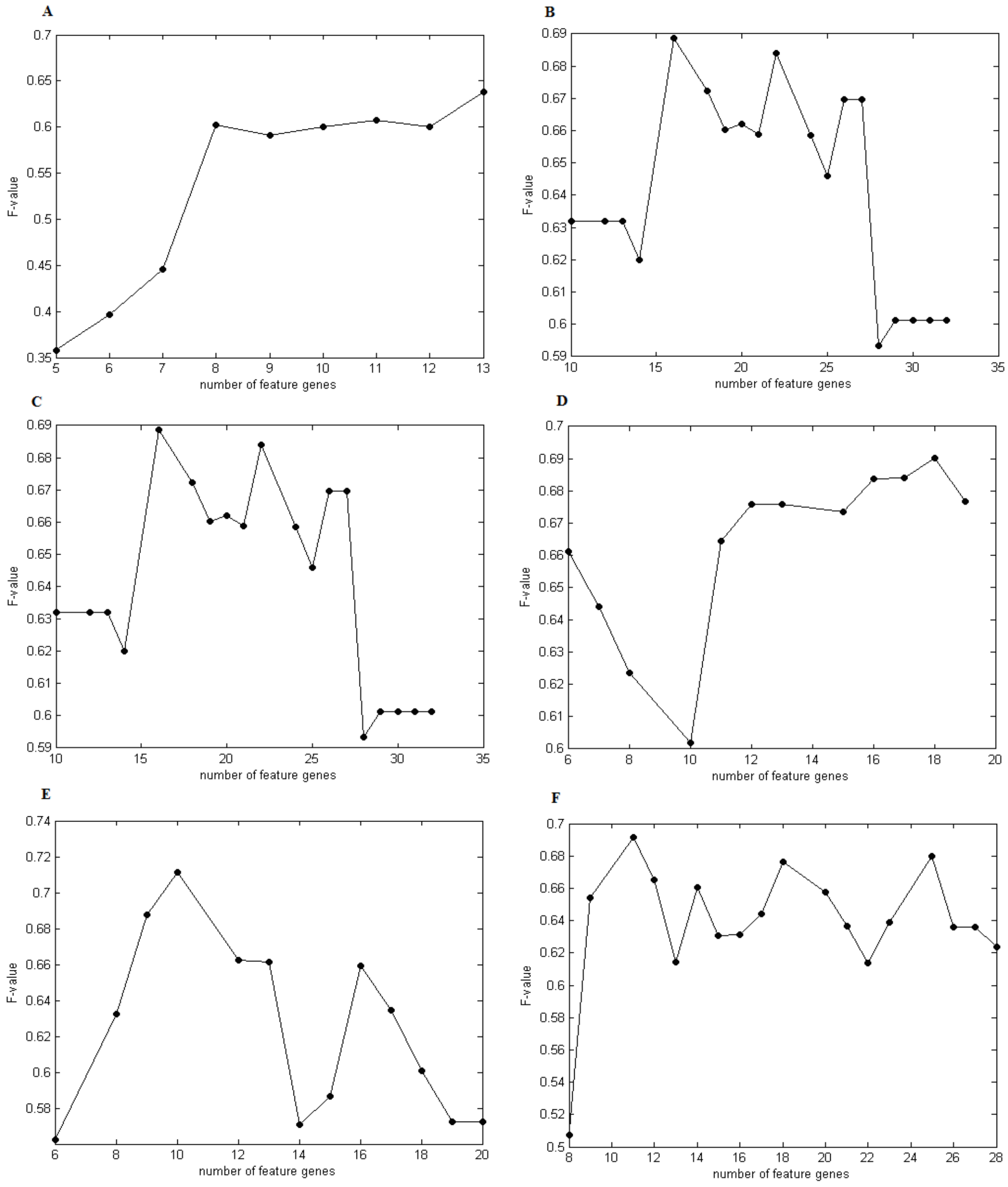

Supplementary Figure 2- Hierarchical clustering of breast tumor samples in GSE22220. (A) mRNA feature genes (the signature), (B) mRNA diff-genes, (C) Sorlie's signature, (D) PAM50 genes.

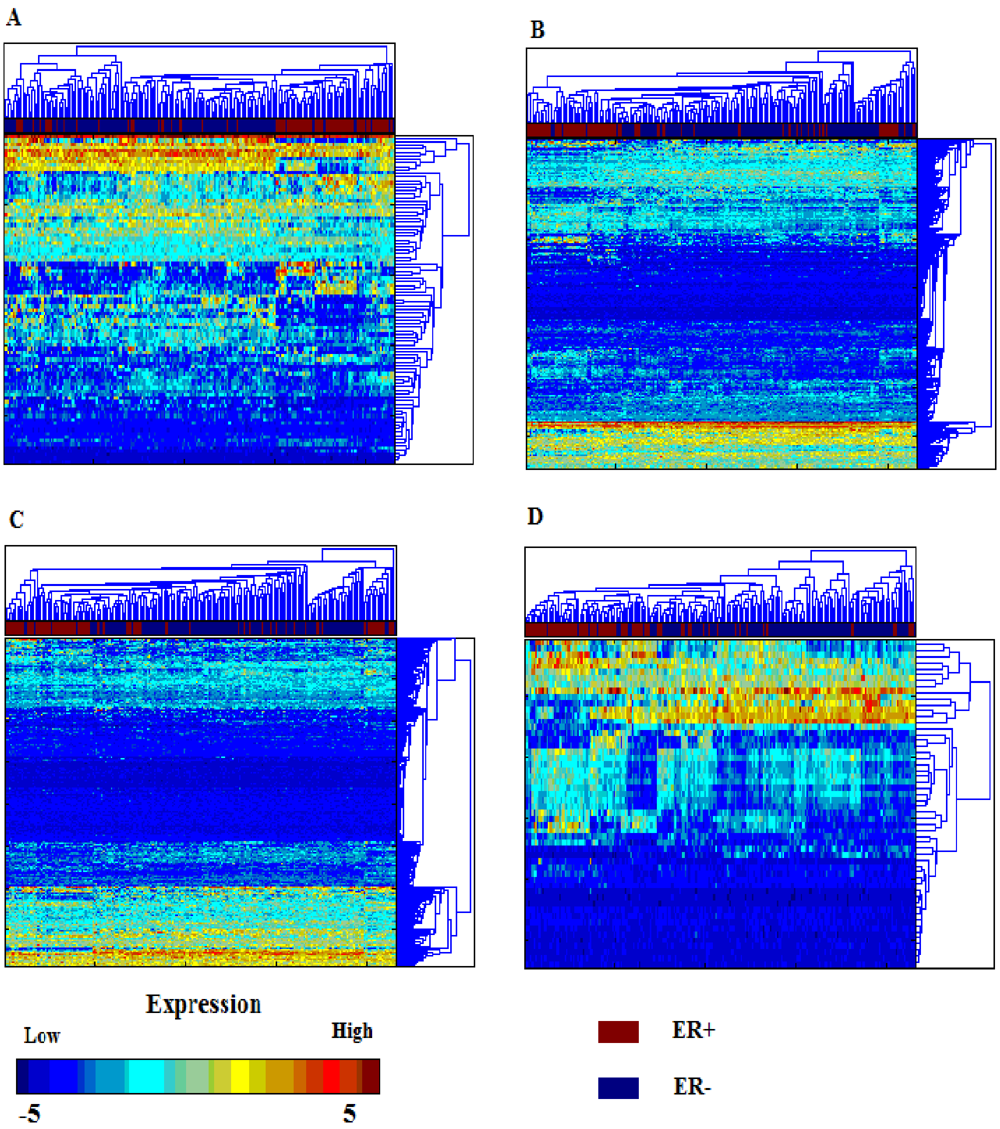

Supplementary Figure 3- Hierarchical clustering of breast tumor samples in TCGA. (A) mRNA feature genes (the signature), (B) mRNA diff-genes, (C) Sorlie's signature, (D) PAM50 genes. Only ER status is available in GSE22220.

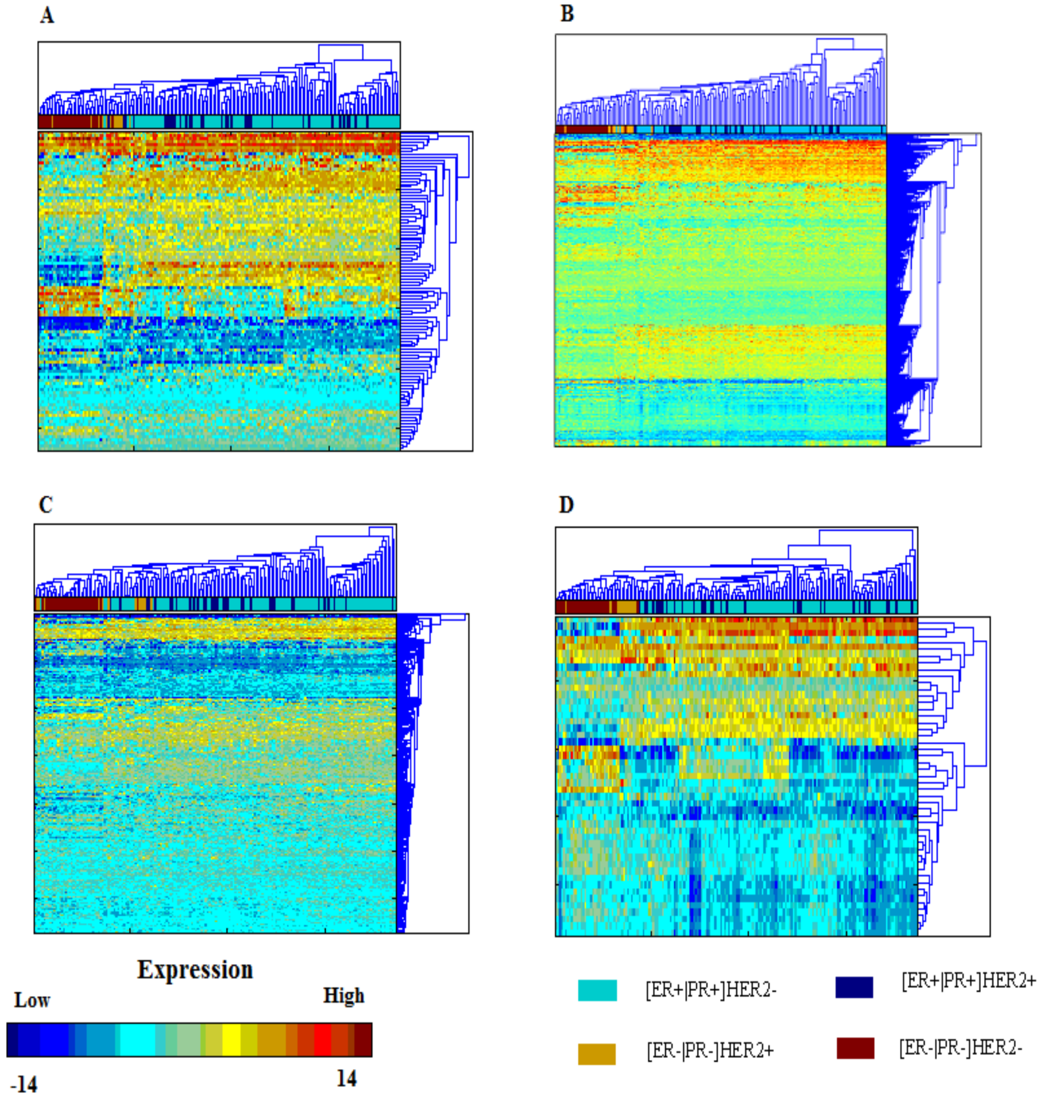

Supplementary Figure 4- Network of subtype-specific feature genes constructed by GeneMANIA. (A) [ER+|PR+]HER2-, (B) [ER+|PR+]HER2+, (C) [ER-|PR-]HER2+, (D) [ER-|PR-]HER2-.

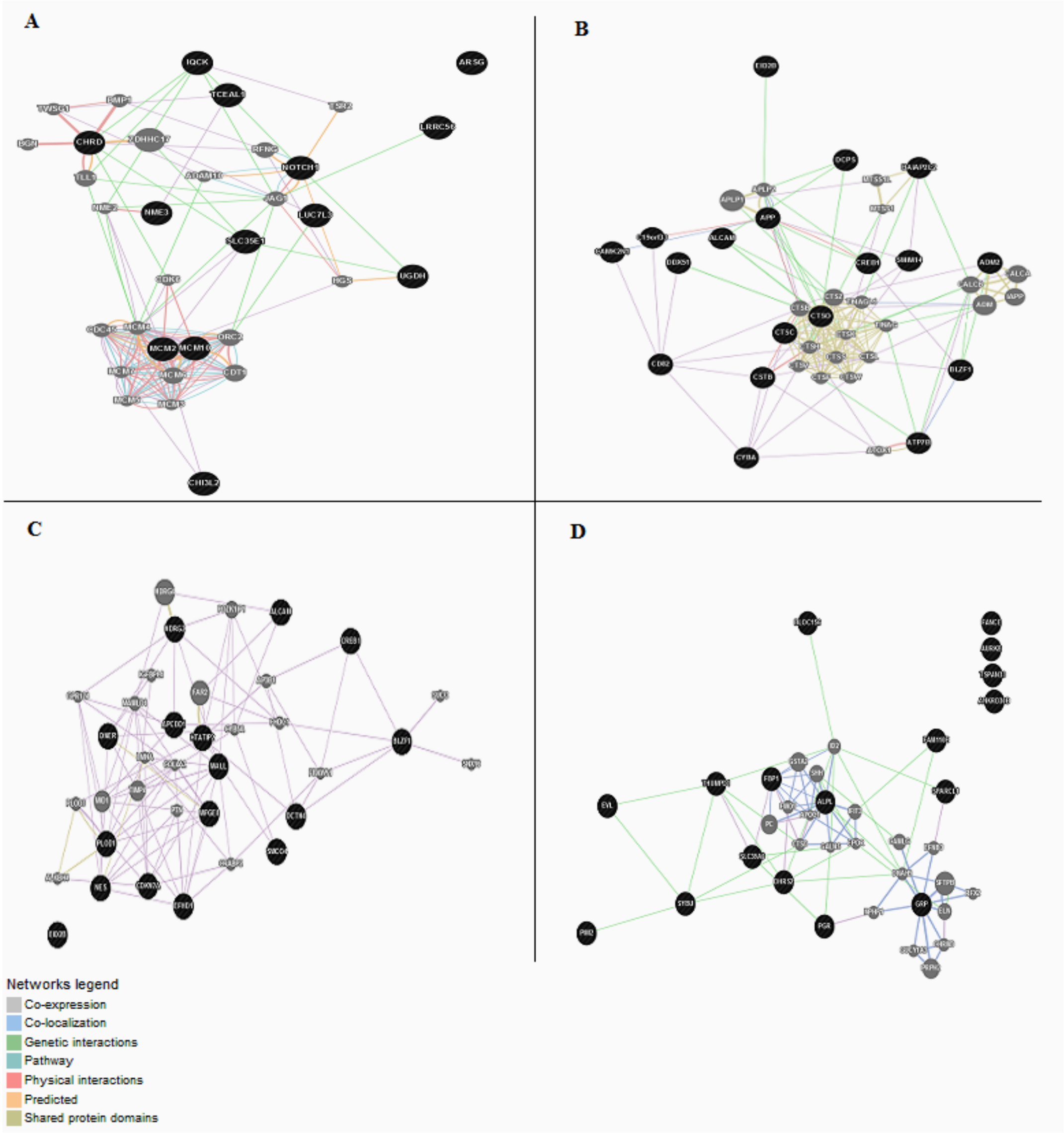

Supplementary Figure 5- Venn diagram illustrating the relationship among the signature, the Sorlie's signature and PAM50 genes.

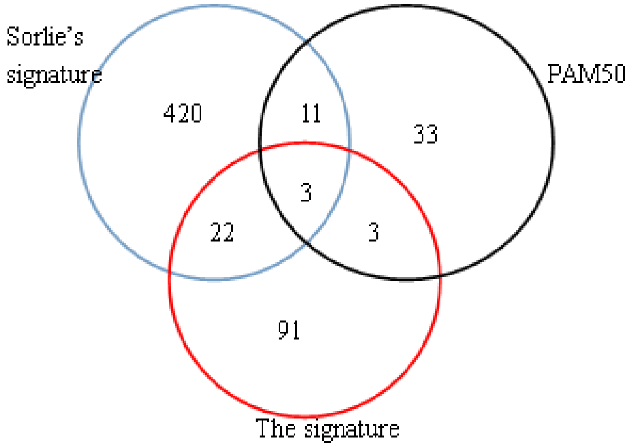

Supplementary table legends

Supplementary Table 1. The signature genes (mRNA feature genes).

Supplementary Table 2. The miRNA feature genes.

Supplementary Table 3. Subtype-specific feature genes.

Supplementary Table 4. KEGG pathways of the signature genes, subtype-specific feature genes and their unified genes.

Supplementary Table 5. KEGG diseases of the signature genes, subtype-specific feature genes and their unified genes.

Supplementary Table 6. Overlap genes between the signature genes and the Sorlie's signature [5] and PAM50 genes [8].

Supplementary Table 1. The signature genes (mRNA feature genes)

S100A9	CILP	MMP7	CD6
S100A8	THBS4	PROM1	CBS
FOXA1	GATA3	C1orf64	ALDH3B2
MLPH	C10orf116	STC2	GLYATL2
GSTO2	CATSPER2	CLIC6	CDCA5
RHBDL2	SFRP1	EEF1A2	KCNF1
AGR3	CRYAB	TFF3	WNK4
ESR1	CGN	TFF1	SYT13
PLA2G16	MAL2	CAPN13	AGTR1
C7orf55	IFI6	VAV3	FSIP1
LAPTM4B	CEBPB	NCCRP1	KIF1A
CCND3	NQO1	CKMT1B	NKX3-1
ANKRD33	PGAP3	DCTN4	CRABP1
PSMB2	KRT17	C14orf104	PPP1R3C
SLC40A1	ACTG2	SLPI	KRT81
FGD3	CAMK2N1	LCN2	C4orf7
ALCAM	SUSD3	GRP	C19orf33
HSD17B4	MUC1	TPSAB1	CCL8
VCAN	CRIP1	TPSG1	SHISA2
SEPT11'	EFHD1	MVK	SERPINA5
STARD10	CRABP2	CLDN8	CLEC3A
SPDEF	SPARCL1	GABRP	GP2
COL1A2	CLDN11	HPGD	PVALB
RPL7P23	FASN	GDF15	HMGCS2
GJA1	COL10A1	STC1	CALML5
NFIX	SCUBE2	COL17A1	DHRS2
SPARC	KRT7	AGR2	TCN1
ZNF652	C9orf152	ELF5	S100A7
MT1X	PHGDH	LOC389033	MUCL1
TBC1D9	ADM	TUBB2B	

Supplementary Table 2. The miRNA feature genes

miRNA symbol	
HS_239	hsa-miR-184
hsa-miR-130b*	hsa-miR-521
hsa-miR-9*	hsa-miR-885-5p
hsa-miR-149	hsa-miR-942
hsa-miR-500*	hsa-miR-411
hsa-miR-135b	hsa-miR-378
hsa-miR-101*	hsa-miR-224
hsa-miR-33b	hsa-miR-375
hsa-miR-449a	hsa-miR-335
hsa-miR-135a	hsa-miR-190b

Supplementary Table 3. Subtype-specific feature genes

Gene type	Gene Symbol	Subtype
mRNA	SLC35E1	1
mRNA	UGDH	1
mRNA	NME3	1
mRNA	CHI3L2	1
mRNA	MCM2	1
mRNA	MCM10	1
mRNA	LRRC56	1
mRNA	NOTCH1	1
mRNA	LUC7L3	1
mRNA	CHRD	1
mRNA	TCEAL1	1
mRNA	ARSG	1
mRNA	IQCK	1
mRNA	CTSC	2
mRNA	CAMK2N1	2
mRNA	ADM2	2
mRNA	C6orf170	2
mRNA	APP	2
mRNA	CSTB	2
mRNA	C4orf34	2
mRNA	BAIAP2L2	2
mRNA	ALCAM	2
mRNA	EID2B	2
mRNA	CREB1	2
mRNA	DDX51	2
mRNA	BLZF1	2
mRNA	ATP7B	2
mRNA	CTSO	2
mRNA	CYBA	2
mRNA	C19orf33	2
mRNA	CD82	2
mRNA	DCPS	2
mRNA	ALCAM	3
mRNA	DCTN4	3
mRNA	DNER	3
mRNA	EID2B	3
mRNA	MALL	3
mRNA	CDKN2A	3
mRNA	MFGE8	3
mRNA	NDRG2	3
mRNA	APCDD1	3
mRNA	EFHD1	3

mRNA	BLZF1	3
mRNA	NES	3
mRNA	PLOD1	3
mRNA	CREB1	3
mRNA	C11orf75	3
mRNA	HTATIP2	3
mRNA	ALPL	4
mRNA	FANCE	4
mRNA	FAM110B	4
mRNA	SLC39A6	4
mRNA	DHRS2	4
mRNA	GRP	4
mRNA	THUMPD1	4
mRNA	RPS4XP16	4
mRNA	AURKB	4
mRNA	PGR	4
mRNA	TSPAN33	4
mRNA	GOLSYN	4
mRNA	PIM2	4
mRNA	SPARCL1	4
mRNA	EVL	4
mRNA	ANKRD30B	4
mRNA	FBP1	4
mRNA	PLDN	4
miRNA	hsa-miR-1295	1
miRNA	hsa-miR-183*	1
miRNA	hsa-miR-184	1
miRNA	hsa-miR-342-5p	1
miRNA	hsa-miR-570	1
miRNA	hsa-miR-135a	2
miRNA	hsa-miR-135b	2
miRNA	hsa-miR-20a*	2
miRNA	hsa-miR-1248	2
miRNA	HS_239	2
miRNA	hsa-miR-106b*	2
miRNA	hsa-miR-1275	2
miRNA	hsa-miR-184	2
miRNA	hsa-let-7f-1*	2
miRNA	hsa-miR-31	2
miRNA	hsa-miR-411	3
miRNA	hsa-miR-663	3
miRNA	hsa-miR-663b	3
miRNA	hsa-miR-130b*	4
miRNA	hsa-miR-135a	4

miRNA	hsa-miR-1295	4
miRNA	hsa-miR-365	4
miRNA	hsa-miR-101*	4
miRNA	hsa-miR-521	4
miRNA	hsa-miR-411	4
miRNA	hsa-miR-33b	4
miRNA	hsa-miR-376a*:9.1	4
miRNA	hsa-miR-181c*	4
miRNA	hsa-miR-31*	4

Subtype:

1: [ER+|PR+]HER2-

2: [ER+|PR+]HER2+

3: [ER-|PR-]HER2+

4: [ER-|PR-]HER2-

Supplementary Table 4. KEGG pathways of the signature genes, subtype-specific feature genes and their unified genes

Term	ID	Input number	Background number	P-Value	Category
Vasopressin-regulated water reabsorption	hsa04962	CREB1;DCTN4	45	0.003071028	subtype1
Huntington's disease	hsa05016	CREB1;DCTN4	183	0.041558582	subtype1
Viral carcinogenesis	hsa05203	CDKN2A;CREB1	206	0.051268192	subtype1
Circadian rhythm	hsa04710	CREB1	31	0.054510572	subtype1
Bladder cancer	hsa05219	CDKN2A	38	0.066042225	subtype1
HTLV-I infection	hsa05166	CDKN2A;CREB1	261	0.077309324	subtype1
Cocaine addiction	hsa05030	CREB1	50	0.085492139	subtype1
Lysine degradation	hsa00310	PLOD1	51	0.087094999	subtype1
Non-small cell lung cancer	hsa05223	CDKN2A	56	0.095068251	subtype1
Dorso-ventral axis formation	hsa04320	NOTCH1	24	0.017575329	subtype2
Ascorbate and aldarate metabolism	hsa00053	UGDH	27	0.019664721	subtype2
Prion diseases	hsa05020	NOTCH1	36	0.025907835	subtype2
Pentose and glucuronate interconversions	hsa00040	UGDH	36	0.025907835	subtype2
DNA replication	hsa03030	MCM2	36	0.025907835	subtype2
Amino sugar and nucleotide sugar metabolism	hsa00520	UGDH	47	0.033487492	subtype2
Notch signaling pathway	hsa04330	NOTCH1	48	0.034173792	subtype2
Starch and sucrose metabolism	hsa00500	UGDH	56	0.039647705	subtype2
TGF-beta signaling pathway	hsa04350	CHRD	80	0.055894776	subtype2
Pyrimidine metabolism	hsa00240	NME3	105	0.072543501	subtype2
Thyroid hormone signaling pathway	hsa04919	NOTCH1	119	0.081745688	subtype2
Lysosome	hsa04142	ARSG	122	0.083706372	subtype2
Cell cycle	hsa04110	MCM2	124	0.085011306	subtype2
Lysosome	hsa04142	CTSC	122	0.015661582	subtype3
Osteoclast differentiation	hsa04380	CREB1;CYBA	131	0.017872203	subtype3
Circadian rhythm	hsa04710	CREB1	31	0.048185192	subtype3
Vasopressin-regulated water reabsorption	hsa04962	CREB1	45	0.068548712	subtype3

Cocaine addiction	hsa05030	CREB1	50	0.075718159	subtype3
Folate biosynthesis	hsa00790	ALPL	14	0.008101664	subtype4
Pentose phosphate pathway	hsa00030	FBP1	28	0.01560851	subtype4
Fructose and mannose metabolism	hsa00051	FBP1	32	0.01774367	subtype4
Fanconi anemia pathway	hsa03460	FANCE	53	0.028883298	subtype4
Acute myeloid leukemia	hsa05221	PIM2	57	0.030991864	subtype4
Glycolysis / Gluconeogenesis	hsa00010	FBP1	66	0.035720695	subtype4
Progesterone-mediated oocyte maturation	hsa04914	PGR	86	0.046153043	subtype4
Carbon metabolism	hsa01200	FBP1	106	0.05648112	subtype4
Oocyte meiosis	hsa04114	PGR	110	0.058534308	subtype4
AMPK signaling pathway	hsa04152	FBP1	124	0.065688029	subtype4
Insulin signaling pathway	hsa04910	FBP1	141	0.074307226	subtype4
Cell adhesion molecules (CAMs)	hsa04514	VCAN;ALCAM;CLDN11;CLDN8;CD6	145	0.004252658	signature
Terpenoid backbone biosynthesis	hsa00900	HMGCS2;MVK	21	0.011317522	signature
Glycine, serine and threonine metabolism	hsa00260	PHGDH;CBS	40	0.035310831	signature
Mineral absorption	hsa04978	SLC40A1;MT1X	51	0.053765569	signature
Leukocyte transendothelial migration	hsa04670	VAV3;CLDN11;CLDN8	118	0.054118168	signature
Vascular smooth muscle contraction	hsa04270	AGTR1;CALML5;ACTG2	121	0.057413585	signature
Focal adhesion	hsa04510	VAV3;COL1A2;THBS4;CCND3	207	0.062386563	signature
Synthesis and degradation of ketone bodies	hsa00072	HMGCS2	9	0.068239078	signature
Tight junction	hsa04530	CLDN11;CGN;CLDN8	134	0.072750462	signature
Ubiquinone and other terpenoid-quinone biosynthesis	hsa00130	NQO1	10	0.07480295	signature
Wnt signaling pathway	hsa04310	MMP7;SFRP1;CC	140	0.080384414	signature

ND3

Insulin signaling pathway	hsa04910	FASN;CALML5;PPP1R3C	141	0.08168943	signature
Drug metabolism - cytochrome P450	hsa00982	GSTO2;ALDH3B2	68	0.087295974	signature
Fatty acid biosynthesis	hsa00061	FASN	13	0.094220026	signature
Prolactin signaling pathway	hsa04917	ELF5; ESR1	72	0.095905861	signature
Lysosome	hsa04142	CTSC;CTSO;ARSG	122	0.009002351	unified
Vasopressin-regulated water reabsorption	hsa04962	CREB1;DCTN4	45	0.011236271	unified
p53 signaling pathway	hsa04115	CDKN2A;CD82	68	0.023869976	unified
Folate biosynthesis	hsa00790	ALPL	14	0.049946749	unified
Cell cycle	hsa04110	CDKN2A;MCM2	124	0.068946148	unified
AMPK signaling pathway	hsa04152	CREB1;FBP1	124	0.068946148	unified
Osteoclast differentiation	hsa04380	CREB1;CYBA	131	0.075705367	unified
Dorso-ventral axis formation	hsa04320	NOTCH1	24	0.081867165	unified
Ascorbate and aldarate metabolism	hsa00053	UGDH	27	0.091235018	unified
Pentose phosphate pathway	hsa00030	FBP1	28	0.094336605	unified
Folate biosynthesis	hsa00790	ALPL	14	0.049946749	unified
Endocrine and other factor-regulated calcium reabsorption	hsa04961	AP2M1;KLK1	48	0.028953591	miRNA targets
Cell cycle	hsa04110	ATM;PRKDC;YWHAZ	124	0.030176084	miRNA targets
Endometrial cancer	hsa05213	AKT2;APC	52	0.033363656	miRNA targets
Signaling pathways regulating pluripotency of stem cells	hsa04550	AKT2;APC;JAK2	142	0.04209244	miRNA targets
mTOR signaling pathway	hsa04150	AKT2;VEGFA	60	0.042903402	miRNA targets
VEGF signaling pathway	hsa04370	AKT2;VEGFA	61	0.044159701	miRNA targets
Colorectal cancer	hsa05210	AKT2;APC	62	0.045429583	miRNA targets
Pancreatic cancer	hsa05212	AKT2;VEGFA	66	0.050641407	miRNA targets

Renal cell carcinoma	hsa05211	AKT2;VEGFA	66	0.050641407	miRNA targets
Hippo signaling pathway	hsa04390	YAP1;APC;YWHAZ	154	0.051161925	miRNA targets
Adipocytokine signaling pathway	hsa04920	AKT2;JAK2	70	0.056056648	miRNA targets
Prolactin signaling pathway	hsa04917	AKT2; JAK2	72	0.058837186	miRNA targets
Non-homologous end-joining	hsa03450	PRKDC	13	0.07174186	miRNA targets
MicroRNAs in cancer	hsa05206	APC; ATM;SOX4;VEGFA	297	0.076541722	miRNA targets
Apoptosis	hsa04210	AKT2;ATM	86	0.079542624	miRNA targets

Category:

subtype1: [ER+|PR+]HER2-

subtype2: [ER+|PR+]HER2+

subtype3: [ER-|PR-]HER2+

subtype4: [ER-|PR-]HER2-

signature: The signature genes

unified: unified subtype-specific feature genes

miRNA targets: validated targets of miRNAs predicted using Mirecords

Supplementary Table 5. KEGG diseases of the signature genes, subtype-specific feature genes and their unified genes

Term	ID	Input number	P-Value	Category
Acute lymphoblastic leukemia (ALL) (Precursor T lymphoblastic leukemia)	H00002	1	0.006048126	subtype1
Cancers of haematopoietic and lymphoid tissues		1	0.026986532	subtype1
Cardiac diseases		1	0.052806888	subtype1
Cancers		1	0.09890529	subtype1
Progressive myoclonic epilepsy (PME)	H00810	1	0.038680988	subtype2
Congenital disorders of ion transport and metabolism		1	0.041715011	subtype2
Lysosomal storage diseases		1	0.075976716	subtype2
Nasopharyngeal cancer	H00054	1	0.027586941	subtype3
Cholangiocarcinoma	H00046	1	0.027586941	subtype3
Chronic myeloid leukemia (CML)	H00004	1	0.031467187	subtype3
Pancreatic cancer	H00019	1	0.031467187	subtype3
Osteosarcoma	H00036	1	0.031467187	subtype3
Laryngeal cancer	H00055	1	0.031467187	subtype3
Malignant melanoma	H00038	1	0.031467187	subtype3
Oral cancer	H00016	1	0.035332417	subtype3
Malignant pleural mesothelioma	H00015	1	0.035332417	subtype3
Bladder cancer	H00022	1	0.039182686	subtype3
Esophageal cancer	H00017	1	0.039182686	subtype3
Non-small cell lung cancer	H00014	1	0.046838571	subtype3
Penile cancer	H00025	1	0.046838571	subtype3
Glioma	H00042	1	0.046838571	subtype3
Hepatocellular carcinoma	H00048	1	0.050644297	subtype3
Ehlers-Danlos syndrome (EDS)	H00802	1	0.050644297	subtype3
Skin cancers		1	0.050644297	subtype3
Cancers of the nervous system		1	0.061973277	subtype3
Head and neck cancers		1	0.061973277	subtype3
Type II diabetes mellitus	H00409	1	0.076874838	subtype3
Cancers of the lung and pleura		1	0.084239278	subtype3
Cancers of endocrine organs		1	0.087900103	subtype3
Fanconi anemia	H00238	1	0.012661115	subtype4
Congenital disorders of cofactor/vitamin metabolism		1	0.016014112	subtype4
Congenital disorders of carbohydrate metabolism		1	0.035105076	subtype4
Congenital disorders of metabolism		2	0.077374959	subtype4
Peroxisomal beta-oxidation enzyme deficiency	H00407	1	0.06376334	signature

Hemochromatosis	H00211	1	0.06376334	signature
Myofibrillar myopathies (MFM)	H00595	1	0.06376334	signature
Metaphyseal dysplasias	H00479	1	0.073992364	signature
Hyperkalemic distal renal tubular acidosis (RTA type 4)	H00243	1	0.073992364	signature
Epidermolysis bullosa, junctional	H00586	1	0.073992364	signature
Vitreoretinal degeneration	H00805	1	0.084110843	signature
Cancers of haematopoietic and lymphoid tissues		2	0.04080226	unified
Nasopharyngeal cancer	H00054	1	0.047907716	unified
Cholangiocarcinoma	H00046	1	0.047907716	unified
Chronic myeloid leukemia (CML)	H00004	1	0.054564901	unified
Pancreatic cancer	H00019	1	0.054564901	unified
Osteosarcoma	H00036	1	0.054564901	unified
Laryngeal cancer	H00055	1	0.054564901	unified
Malignant melanoma	H00038	1	0.054564901	unified
Oral cancer	H00016	1	0.061176335	unified
Malignant pleural mesothelioma	H00015	1	0.061176335	unified
Acute lymphoblastic leukemia (ALL) (Precursor T lymphoblastic leukemia)	H00002	1	0.067742328	unified
Bladder cancer	H00022	1	0.067742328	unified
Esophageal cancer	H00017	1	0.067742328	unified
Non-small cell lung cancer	H00014	1	0.080739211	unified
Penile cancer	H00025	1	0.080739211	unified
Glioma	H00042	1	0.080739211	unified
Hepatocellular carcinoma	H00048	1	0.08717071	unified
Ehlers-Danlos syndrome (EDS)	H00802	1	0.08717071	unified
Skin cancers		1	0.08717071	unified
Fanconi anemia	H00238	1	0.099901326	unified

Category:

subtype1: [ER+|PR+]HER2-

subtype2: [ER+|PR+]HER2+

subtype3: [ER-|PR-]HER2+

subtype4: [ER-|PR-]HER2-

signature: The signature genes

unified: unified subtype-specific feature genes

Supplementary Table 6. Overlap genes between the signature genes and the Sorlie's signature [5] and PAM50 genes [8]. There are 25 genes shared between the signature and Sorlie's signature (Sorlie&PAM50, Sorlie), 6 genes overlapping between the signature and PAM50 genes (Sorlie&PAM50, PAM50). Among them 3 genes are shared among all the three gene sets (Sorlie&PAM50).

Sorlie&PAM50	ESR1	FOXA1	KRT17
Sorlie	S100A8	GATA3	KRT7
	SLC40A1	ALCAM	NFIX
	CRYAB	IFI6	CRABP1
	CRABP2	FASN	COL17A1
	SLPI	STC1	CAMK2N1
	PLA2G16	LAPTM4B	TFF3
	MT1X	PVALB	MUC1
	CKMT1B		
PAM50	SFRP1	MLPH	PHGDH

Supplementary references

[5]Sorlie, T. *et al.* Gene expression patterns of breast carcinomas distinguish tumor subclasses with clinical implications. *PNAS* **98**, 10869-10874, doi:10.1073/pnas.191367098 (2001).

[8] Parker, J. S. *et al.* Supervised risk predictor of breast cancer based on intrinsic subtypes. *JCO* **27**, 1160-1167, doi:10.1200/JCO.2008.18.1370 (2009).
